# Differential responses of blood-brain barrier associated cells to hypoxia and ischemia: a comparative study

**DOI:** 10.1186/2045-8118-12-4

**Published:** 2015-02-17

**Authors:** Sabrina Engelhardt, Sheng-Fu Huang, Shalmali Patkar, Max Gassmann, Omolara O Ogunshola

**Affiliations:** Institute of Veterinary Physiology & Zurich Center for Integrative Human Physiology (ZIHP), Vetsuisse Faculty, University of Zurich, Winterthurerstrasse 260, CH-8057 Zurich, Switzerland

**Keywords:** Endothelial cells, Astrocytes, Pericytes, Cell death, HIF-1, *in vitro*

## Abstract

**Background:**

Undisturbed functioning of the blood–brain barrier (BBB) crucially depends on paracellular signaling between its associated cells; particularly endothelial cells, pericytes and astrocytes. Hypoxic and ischemic injuries are closely associated with disturbed BBB function and the contribution of perivascular cells to hypoxic/ischemic barrier regulation has gained increased attention. Regardless, detailed information on the basal hypoxic/ischemic responses of the barrier-associated cells is rare and the outcome of such cell-specific responses on BBB modulation is not well understood. This study investigated crucial parameters of hypoxic/ischemic adaptation in order to characterize individual perivascular cell responses to stress conditions.

**Methods:**

The brain microvascular endothelial cell line RBE4 (EC cell line) as well as primary rat brain endothelial cells (ECs), pericytes (PCs) and astrocytes (ACs) were exposed to 24 and 48 hours of oxygen deprivation at 1% and 0.2% O_2_. All primary cells were additionally subjected to combined oxygen and glucose deprivation mimicking ischemia. Central parameters of cellular adaptation and state, such as HIF-1α and HIF-1 target gene induction, actin cytoskeletal architecture, proliferation and cell viability, were compared between the cell types.

**Results:**

We show that endothelial cells exhibit greater responsiveness and sensitivity to oxygen deprivation than ACs and PCs. This higher sensitivity coincided with rapid and significant stabilization of HIF-1α and its downstream targets (VEGF, GLUT-1, MMP-9 and PHD2), early disruption of the actin cytoskeleton and metabolic impairment in conditions where the perivascular cells remain largely unaffected. Additional adaptation (suppression) of proliferation also likely contributes to astrocytic and pericytic tolerance during severe injury conditions. Moreover, unlike the perivascular cells, ECs were incapable of inducing autophagy (monitored via LC3-II and Beclin-1 expression) - a putative protective mechanism. Notably, both ACs and PCs were significantly more susceptible to glucose than oxygen deprivation with ACs proving to be most resistant overall.

**Conclusion:**

In summary this work highlights considerable differences in sensitivity to hypoxic/ischemic injury between microvascular endothelial cells and the perivascular cells. This can have marked impact on barrier stability. Such fundamental knowledge provides an important foundation to better understand the complex cellular interactions at the BBB both physiologically and in injury-related contexts *in vivo.*

## Background

Brain neurons require a stable environment and high nutrient supply for proper functioning [1]. In this regard, the well-coordinated teamwork of the cells at the blood–brain barrier (BBB) is indispensable. Endothelial cells that line barrier vessels have intimate contact with supporting pericytes and astrocytes, as well as microglia, neurons and a well-defined extracellular matrix to fulfill their gatekeeper function at the BBB, crucially controlling nutrient supply and homeostatic balance [2]. It has become very apparent that these endothelial cells depend on inductive signaling of the surrounding cell types to fully develop and maintain their unique barrier phenotype [3, 4]. Recently the important contribution of perivascular astrocytes and pericytes to BBB induction and maintenance has received increasing attention (for review see [5]).

Hypoxia (reduced tissue oxygenation) severely impairs BBB function *in vivo* and *in vitro*[6–8] causing pathophysiological changes including disturbed energy balance and water/ion homeostasis [5], inflammatory events and leakage of blood-proteins into the brain [5]. Similarly ischemia, being characterized by impaired nutrient and O_2_ supply, also results in significant BBB breakdown [9, 10]. Clearly, during injury conditions specific responses of astrocytes and pericytes impact BBB maintenance and brain function. Both astrocytes and pericytes have been shown to support barrier function via tight junction retention and reduction of endothelial cell death under hypoxic conditions [6, 11–14]. On the contrary, both cell types up-regulate barrier permeabilizing factors in response to hypoxia/ischemia such as vascular endothelial growth factor (VEGF), cytokines, and matrix metalloproteinases (MMPs) [15]. Currently we do not understand well how, or from where, the differential signals that alter BBB characteristics originate, although some evidence suggests that injury severity and duration determines the functional outcome of hypoxic/ischemic insults on BBB tightness [5, 16, 17]. In this regard hypoxia-inducible factor 1 (HIF-1), a central regulator of hypoxic/ischemic responses, is of particular importance. HIF-1 constitutes a heterodimeric transcription factor composed of the hypoxia-inducible HIF-1α subunit and the constitutively expressed HIF-1β subunit [18]. HIF-1 mediates hypoxic adaptation through transcriptional control of more than 100 genes participating in angiogenesis, proliferation and cellular metabolism to reduce O_2_ consumption and increase O_2_ supply but can also trigger cell death [18, 19]. Notably, the ability of different cell types to adapt to hypoxic/ischemic stress varies considerably. Neurons for example are very sensitive to such insults, whereas other brain cells such as astrocytes are far more resistant [1]. Our previous study showed that astrocytes and pericytes differentially modulate BBB permeability at varying severities of hypoxic insults [6], implying that insult severity likely triggers individual cell-specific responses with subsequent consequences on barrier modulation.

To gain better knowledge of cell-specific behavior in terms of overall tolerance as well as potential signaling to surrounding cells, we directly compared basal responses of individual BBB associated cells (brain microvascular endothelial cell line and primary rat endothelial cells (ECs), pericytes (PCs) and astrocytes (ACs) to hypoxic and ischemic insults *in vitro*. We analyzed different parameters that influence hypoxic and ischemic responses including HIF-1α stabilization and target gene expression, actin cytoskeletal alterations, proliferation, and cell survival. Notably the responsiveness and tolerance of the three cell types differed considerably and likely underlies their ability to maintain and/or modulate BBB integrity.

## Material and methods

### Cell culture and isolation of primary cells

All cell culture media and reagents were obtained from Gibco® (Life Technologies, Zug, Switzerland). A rat brain endothelial cell line, RBE4 (EC cell line) [20] was cultivated from passage 35–45 in 1:1 α-MEM/Ham’s F-10 medium mixture supplemented with 10% FBS, 300 μg/ml geneticin, and 1 ng/ml basic fibroblast growth factor (Pepro Tech, Rocky Hill, NJ, USA) on rat tail collagen coated petri dishes. Rat tail collagen was isolated as described in Roll *et al.*[21]. Primary rat brain endothelial cells (primary ECs) were isolated from 8–10 week old male Wistar rats according to Coisne *et al.*[22], with slight modifications. Isolated cortices were homogenized after removal of meninges in a 40 ml Dounce homogenizer. Microvessels were isolated from the homogenate by adding an equal volume of 30% Dextran solution and subsequent centrifugation at 3000 g for 25 min at 4°C. The microvessels were then filtered through a 60 μm nylon mesh to remove larger vessels and digested in HBSS buffer supplemented with 10 mM HEPES, 0.1% BSA, 2 mg/ml collagenase-dispase, 10 μg/ml DNAse I and 147 ng/ml TLCK, for 45 min at 37°C in a water bath. Digested microvessels were re-suspended in endothelial media (DMEM Glutamax II supplemented with 10& calf serum, BME amino acids, vitamin solution, 2 mM L-glutamine, 1 ng/ml bFGF, 50 μg/ml gentamycin sulfate) and plated on collagen-IV (Sigma-Aldrich)-coated culture dishes. One day after isolation primary ECs were treated with 3 μg/ml puromycin-containing media for 24 h to prevent pericyte contamination and then used in experiments on day 5 after isolation (i.e. without passaging). Primary rat astrocytes (ACs) were isolated from neonatal pups as described previously [23] and cultured in DMEM supplemented with 10% FBS and 50 μg/ml gentamycin sulfate. Primary rat brain pericytes (PCs) were isolated from male adult rats following an established protocol [24] with slight modifications. Briefly, cortices were homogenized after removal of meninges. Subsequently microvessels were isolated by centrifugation (20 min at 3000 g at 4°C) using dextran (final concentration 16%) and digested in DMEM + 0.1% Collagenase/Dispase + 125 ug/ml DNAse I (both Roche Diagnostics, Rotkreuz, Switzerland) in a water bath for 2 h at 37°C. After centrifugation (700 g, 5 min) freshly isolated pericytes were plated on rat tail collagen coated dishes and cultured in DMEM supplemented with 20% FBS, 50 μg/ml gentamycin sulfate and 2.5 μg/ml amphotericin B until confluency. Subsequent passages were maintained on uncoated dishes in astrocyte medium. For all experiments astrocytes and pericytes were used at passage 2. The purity of all primary cultures was ≥95% as analyzed by immunostaining for standard cell markers namely GFAP (astrocytes), PECAM-1 (endothelial cells), NG-2 (pericytes) and PDGFR-β (pericytes).

### Hypoxic and ischemic exposures

O_2_ deprivation experiments were carried out in purpose-built hypoxic glove-box chambers (InVivO_2_ 400, Ruskinn Technologies, Pencoed, UK) maintained at 37°C with 5% CO_2_. O_2_ concentration was constantly monitored with an internal O_2_ sensor. Cells were exposed to hypoxia (1% O_2_) and near anoxia (0.2% O_2_) for up to 48 h. Normoxic controls were maintained at 21% O_2_, 5% CO_2_ at 37°C. Ischemia was simulated *in vitro* by oxygen-glucose deprivation (OGD). OGD exposures were carried out on all primary cells under hypoxia and near anoxia using glucose-free media.

### Western blotting

Cells were washed with ice-cold PBS and homogenized in cell lysis buffer (50 mM Tris, 150 mM NaCl, 1% Triton X-100, 1% NP-40) supplemented with protease inhibitor cocktail (Calbiochem, Darmstadt, Germany), 1 mM sodium orthovanadate, 1 mM dithiothreitol, 0.5 mM phenylmethansulfonyl fluoride and 1 mM EDTA. Protein concentration was determined with Pierce BCA protein assay (Thermo Fisher Scientific Inc., Rockford, IL, USA). Total proteins (20 μg) were separated on denaturing SDS-Page and transferred onto a nitrocellulose membrane. Membranes were blocked at room temperature in 5% non-fat dried milk or 5% BSA dissolved in Tris-buffered saline containing 0.1% Tween-20 and subsequently incubated overnight at 4°C with primary antibodies against β-actin (1:5000, Sigma–Aldrich, Buchs, Switzerland), α-tubulin (1:2000, Sigma–Aldrich), HIF-1α (1:1000, Novus Biologicals, Littleton, CO, USA), LC3 (1:2000, Novus Biologicals), Beclin-1 (1:250, Santa Cruz Biotech, Heidelberg, Germany), Bax (1:1000, Merck Milipore, Darmstadt, Germany) or BNIP3 (1:1000, Cell Signaling Technology, Leiden, The Netherlands). Membranes were washed with 0.1% Tween-20 in TBS then incubated with horseradish peroxidase conjugated secondary antibody (ImmunoResearch, Suffolk, UK). Band detection was performed and visualized using a luminescent image analyzer *LAS*-*3000* (Fujifilm, Dielsdorf, Switzerland). Blot quantification (using β-actin and α-tubulin as loading controls) was performed using ImageJ software (ImageJ, NIH, Bethesda, USA).

### Quantitative real-time PCR

Total RNA was isolated directly from culture dishes using TRIzol® Reagent (Life Technologies, Zug, Switzerland) according to the manufacturer`s description. One μg of RNA per sample was reverse transcribed using the ImProm-II ReverseTranscriptase kit (Promega, Dübendorf, Switzerland) according to the manufacturer’s instructions.

Quantitative real-time PCR was performed with an ABI 7500 Fast Real-Time PCR System (Applied Biosystems, Zug, Switzerland) using Power Sybr® Green PCR Master Mix (Applied Biosystems). The following primers at 0.2 μm final concentration were used: PHD2 5′-AAGCCATGGTCGCCTGTTAC-3′ and 5′-TGCGTACCTTGTGGCGTATG-3′, VEGF 5′-CGCAAGAAATCCCGGTTTAA-3′ and 5′-CAAATGCTTTCTCCGCTCTGA-3′, GLUT-1 5′-GGGCATGATTGGTTCCTTCTC-3′ and 5′-CAGGTTCATCATCAGCATGGA-3′, MMP-9 5′-GGGAACGTATCTGGAAATTCGAC-3′ and 5′-CCGGTTGTGGAAACTCACAC-3′, BNIP3 5′-GCTCCCAGACACCACAAGA-3′ and 5′-GCTGAGAAAATTCCCCCTTT-3′ and β-actin 5′-CTGGCTCCTAGCACCATGAAG-3′ and 5′-GCCACCGATCCACACAGAGT-3′. For each cell type, a five-fold dilution series was prepared from the cDNA and standard curves were constructed separately for each target gene. PCR efficiencies were calculated from the standard curve slopes for all primer sets. This resulted in 90-100% efficiency for all targets measured. Furthermore, a single band of the expected size for each target, without primer dimers or off-target amplifications, was confirmed by gel electrophoresis (data not shown). All data were normalized to β-actin. Fold changes were calculated based on the comparative ΔΔCt method.

### F-actin staining and microscopy

The EC cell line was grown on rat tail collagen coated coverslips, primary ECs were grown on coverslips coated with commercially available collagen IV, ACs on gelatin-coated and PCs on uncoated coverslips until confluency. After hypoxic and ischemic exposure cells were fixed in 4% paraformaldehyde, permeabilized in 0.1% Triton X-100 in PBS and stained for filamentous actin (F-actin) using rhodamine-conjugated phalloidin (Life Technologies). Cell nuclei were counterstained with DAPI (4′,6-Diamidin-2-phenylindol). Pictures were taken using an inverted fluorescence microscope coupled to an 8-bit CCD camera (Axiocam HR, Carl Zeiss, Feldbach, Switzerland) and processed using ImageJ software.

### BrdU incorporation assay

Cell proliferation was measured via BrdU incorporation using the colorimetric BrdU Cell Proliferation ELISA (Roche Diagnostics) according to the manufacturer’s instructions. Briefly, 80-90% confluent primary ECs, ACs and PCs were subjected to OGD and O_2_ deprivation for 24 h and 48 h. For 24 h treatments BrdU reagent was added simultaneously with onset of OGD/O_2_ deprivation, whereas for 48 h exposures BrdU was added 24 h before the end of the treatment. After exposure cells were fixed for 30 min and incubated for 1.5 h at 37°C with a peroxidase conjugated BrdU antibody (anti-BrdU-POD). After three PBS washes, peroxidase substrate was added and plates incubated to reach an OD_405/520_ of 0.4-0.5. The colorimetric reaction was terminated by adding 1 M H_2_SO_4_ and absorbance was read at 450/620 nm using a Multiskan RC Microplate Photometer (Thermo Labsystems, Helsinki, Finland).

### MTT assay

Cell viability was measured using MTT (3-(4,5-dimethylthiazol-2-yl)-2,5-diphenyltetrazolium bromide) assay. Briefly all cells were cultured in 96-well plates. After ischemic/hypoxic exposures MTT solution (Sigma-Aldrich) was added to the medium to a final concentration of 0.5 mg/ml and the plates were incubated for 1 h at 37°C. Subsequently the media was removed and cells were lysed by adding equal volume of dimethyl sulfoxide. Optical density was measured at 560/670 nm using a Multiskan RC Microplate Photometer (Thermo Labsystems).

### Statistical analysis

All results are expressed as mean ± SD of three or more independent experiments. Statistical significance was assessed by one-way ANOVA for comparison within one group or two-way ANOVA for comparison between different groups using GraphPad Prism 5 software (La Jolla, CA). Bonferroni’s post hoc test was used for the majority of analyses. Kolmogorov–Smirnov test was used to assess the qPCR data for normal distribution and subsequently the Kruskal-Wallis non-parametric test was used to evaluate significance. A *P*-value <0.05 was considered significant.

## Results

### HIF-1α induction profiles in BBB associated cells

**Figure 1:**
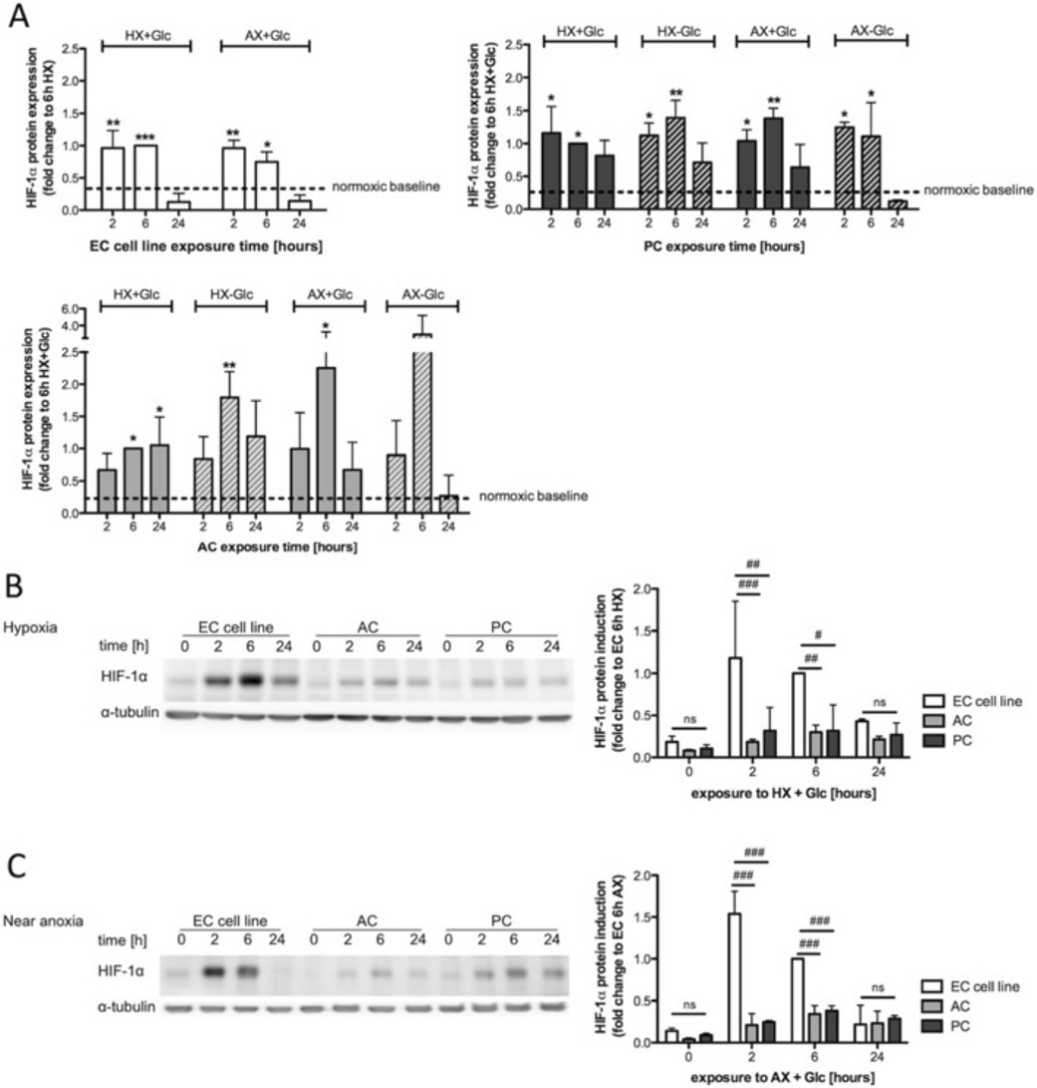
**HIF-1α induction profiles in barrier associated cells.** Western blot analysis of HIF-1α protein expression in EC cell line, PCs and ACs exposed to HX (1% O_2_) and AX (0.2% O_2_) in presence or absence of glucose for up to 24 h. **(A)** Densitometric quantification of HIF-1α protein expression in EC (upper left panel), PC (upper right panel) and AC (lower left panel). N = 3; *P < 0.05, **P < 0.01, ***P < 0.001; 1-way ANOVA compared to normoxic baseline. Representative comparative Western blots of HIF-1α expression in different cell types and their densitometric quantifications after exposure to hypoxia **(B)** and near anoxia **(C)**. N = 3. ^#^ *P* < 0.05, ^##^ *P* < 0.01, ^###^ *P* < 0.001; 2-way ANOVA compared to EC cell line. EC cell line: RBE4, AC: astrocyte, PC: pericyte, HX: hypoxia, AX: near anoxia, +Glc: with glucose, -Glc: without glucose.

Stabilization of HIF-1α protein levels in response to O_2_ deprivation is a central mechanism by which cells adapt to reduced oxygenation [25]. HIF-1α expression profiles of EC cell line, ACs and PCs were analyzed after exposure to hypoxia (HX, 1% O_2_) or near anoxia (AX, 0.2% O_2_) for up to 24 h and compared to normoxic baseline expression (NX + Glc) indicated by the dashed line (Figure 1A). In addition, ACs and PCs were subjected to oxygen-glucose deprivation (OGD) at 1% and 0.2% O_2._ Since the EC cell line was extremely sensitive to O_2_ deprivation alone and OGD led to significant cell death, additional OGD treatment was not performed. In all cell types HIF-1α protein was rapidly stabilized within 2 to 6 h of exposure and then reduced again after 24 h (Figure 1A). Notably the induction profiles differed across the cell types; in the EC cell line HIF-1α levels peaked already at 2 h whereas in ACs peak induction occurred at 6 h. Moreover, AC and PC HIF-1α levels remained elevated after 24 h for most exposures, whereas expression levels in EC cell line had already returned to baseline. Importantly, the EC cell line and PCs did not show any large difference in total HIF-1α expression between HX and AX at any time point and similarly OGD had no additional effect on HIF-1α stabilization in PCs compared to O_2_ deprivation alone. In contrast HIF-1α expression was clearly increased in ACs depending on the severity of insult i.e. HX versus AX and O_2_ deprivation versus OGD (Figure 1A). To directly compare the amount of HIF-1α protein across the three cell types, equal amounts of protein from cells exposed to HX (Figure 1B) or AX (Figure 1C) in presence of glucose, were simultaneously analyzed on the same Western blot membrane. Intriguingly the EC cell line consistently showed 3–5 times higher HIF-1α expression than ACs and PCs.

### Differential regulation of HIF-1 and ischemia-inducible gene expression in BBB associated cells

**Figure 2:**
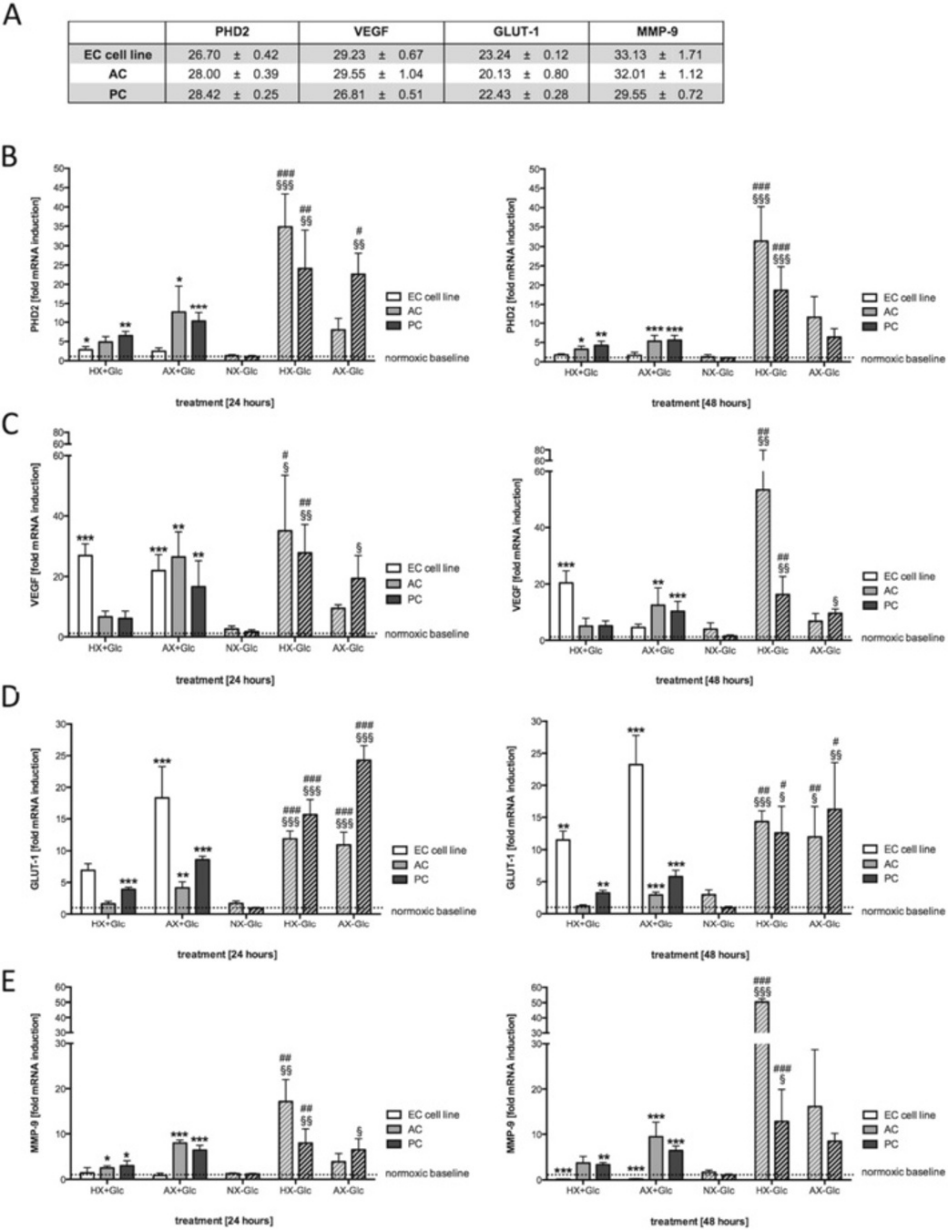
**Differential regulation of HIF-1 target genes in barrier associated cells by quantitative PCR. (A)** Table of basal normoxic expression of the analyzed genes showing threshold cycles (CT) ± standard deviation. mRNA expression profiles of PHD2 **(B)**, VEGF **(C)**, GLUT-1 **(D)** and MMP-9 **(E)** after 24 h (left panel) and 48 h (right panel) of O_2_ deprivation (solid bars) and oxygen glucose deprivation (hatched bars). N = 3-4. *P < 0.05, **P < 0.01, ***P < 0.001; 1-way ANOVA compared to normoxic baseline. ^§^ *P* < 0.05, ^§§^ *P* < 0.01, ^§§§^ *P* < 0.001; 1-way ANOVA compared to normoxia-glucose. ^#^ *P* < 0.05, ^##^ *P* < 0.01, ^###^ *P* < 0.001; 2-way ANOVA comparing with (+) to without (-) glucose treatments. EC cell line: RBE4, AC: astrocyte, PC: pericyte, NX: normoxia, HX: hypoxia, AX: near anoxia, +Glc: with glucose, -Glc: without glucose, PHD2: prolyl hydroxylase 2, VEGF: vascular endothelial growth factor, GLUT-1: glucose transporter 1, MMP-9: matrix metalloproteinase 9.

To further characterize the individual BBB cell responses to reduced oxygenation, mRNA expression profiles of the HIF-1 targets PHD2 (prolyl hydroxylase 2), VEGF and GLUT-1 as well as the hypoxia/ischemia-inducible MMP-9 [26] were analyzed by quantitative real-time PCR after 24 and 48 h of treatment (Figure 2). A table of the basal normoxic threshold cycles (CT) for the individual cell types and analyzed genes is presented in Figure 2A. The EC cell line had the highest basal expression of PHD2 (CT = 26.7) compared to ACs and PCs (CT = 28) (Figure 2A). The cell line showed only weak 3-fold induction during O_2_ deprivation after 24 h compared to the perivascular cell induction of 5 and10-fold after 24 h HX and AX respectively (Figure 2B). Interestingly, HX-Glc exposure led to a further pronounced increase in PHD2 mRNA in ACs and PCs, whereas induction after 24 h AX-Glc was only observed in PCs. Generally the PHD2 profiles after 48 h were similar albeit with slightly lower induction levels and loss of the strong PC induction under AX-Glc.

Different responses were observed for VEGF induction (Figure 2C). Here PCs had the highest basal expression during NX (CT = 26.8) whereas EC cell line and ACs had values of about 29 (Figure 2A). After 24 h of HX EC cell line showed a much stronger induction than ACs and PCs whereas during AX the VEGF mRNA levels were comparable between all cell types (Figure 2C), again suggesting the EC cell line reacts more strongly and rapidly to O_2_ deprivation. Notably OGD induced a stronger increase in VEGF induction in ACs and PCs. Again, expression patterns at 48 h were similar to 24 h. A gradual increase in basal GLUT-1 expression was observed from EC cell line (CT 23) over PCs (CT 22) to ACs (CT 20; Figure 2A). In all cell types, increased GLUT-1 mRNA expression correlated with increased severity and duration of the insult with the EC cell line displaying the strongest induction (up to 20 fold) during O_2_ deprivation (Figure 2D). Similar to our findings with VEGF, OGD was a stronger inducer of GLUT-1 mRNA in ACs and PCs compared to O_2_ deprivation alone.

Interestingly, basal expression of MMP9 mRNA (CT 33, Figure 2A) was very low in the EC cell line, unaltered after 24 h of O_2_ deprivation and even decreased after 48 h (Figure 2E). ACs and PCs showed comparable severity-dependent up-regulation of MMP9 mRNA after 24 and 48 h in presence of glucose and OGD differed only in HX-Glc where ACs showed a remarkable increase of MMP-9 compared to PCs.

Notably, tests on primary ECs for basal expression of the above targets revealed similar CT values compared to the cell line (data not shown) highlighting similar responses of the cell line and primary cells. In conclusion, these findings underline the differential regulation of hypoxic/barrier-modulating genes in cells at the BBB and the consequence of cell-specific responses.

### Injury induced actin cytoskeletal rearrangements in BBB associated cells

**Figure 3:**
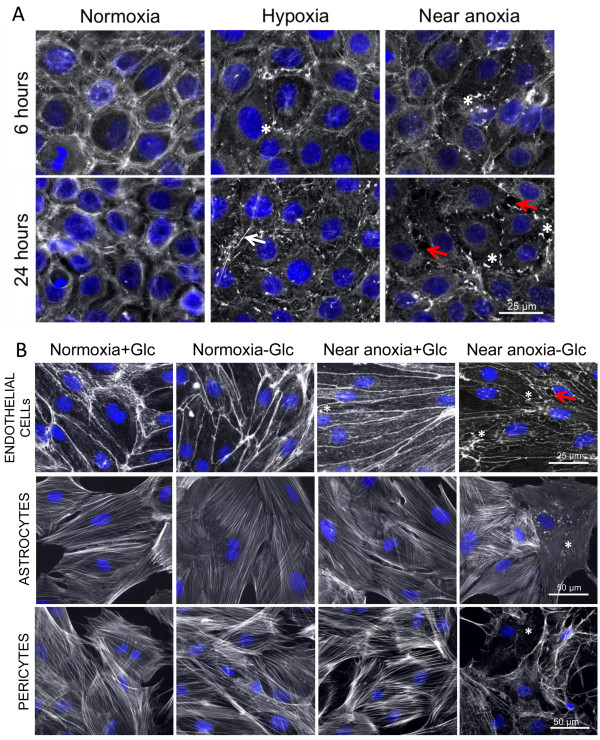
**O**_**2**_ **deprivation and ischemia induce actin cytoskeleton rearrangements in barrier associated cells.** F-actin fibers stained with phalloidin (white) and cell nuclei with DAPI (blue). **(A)** Monolayers of EC cell line exposed to normoxia, hypoxia and near anoxia for 6 and 24 h. **(B)** F-actin staining of primary endothelial cell (upper panel), astrocytes (middle panel) and pericytes (lower panel) exposed to normoxia and near anoxia in presence or absence of glucose for 24 h. White arrow shows stress fiber formation; red arrows indicate inter-endothelial gap formation. Asterisk highlights breakdown of F-actin resulting in punctuate staining.

Reduced oxygenation causes dynamic reorganisation of the actin cytoskeleton resulting in changes of cell contractility, shape and motility [27] thereby providing important information on cell state and activation. Thus we monitored alterations in F-actin organisation under different conditions via phalloidin staining, and used such changes to make conclusions of the sensitivity of our cells to injury (Figure 3). Under normoxic conditions, parallel bundles of F-actin were observed in both the EC cell line (Figure 3A) and primary ECs (Figure 3B) that either localized to cell-cell borders and/or formed a cortical actin rim, structures crucial for maintaining endothelial tightness [28]. In the EC cell line, within 6 h HX- and AX-mediated disruption of the cortical actin rim was observed and characterized by disassembled F-actin (asterisk, Figure 3A). After 24 h of either treatment the cortical actin rims were completely disassembled, suggesting major disruption of endothelial integrity (white arrow). Furthermore large inter-endothelial gaps were observed after 24 h of AX (red arrows, Figure 3A) and by 48 h of AX a large number of cells were detached or dead (data not shown). Using primary ECs 24 h of HX (data not shown) and AX also caused disorganisation of the actin cytoskeleton in the presence of glucose (asterisk, Figure 3B) compared to normoxic controls. In HX-Glc, this disruption was further characterised by condensation of actin near the membrane (data not shown) and such observations were exaggerated by 24 h of Ax-Glc with the formation of inter-endothelial gaps (red arrow, Figure 3B) - suggesting major disruption of endothelial integrity.

ACs and PCs have a different F-actin architecture with parallel bundles arranged across the cells in so called stress fibers [28]. In contrast to the EC cell line and EC primary cultures, both ACs and PCs proved to be very insensitive to O_2_ deprivation in presence of glucose as no alterations in F-actin organization were observed after 24 h in either HX (data not shown) or AX (Figure 3B) and even prolonged treatment for 48 h had no effect (data not shown). Although HX-Glc was ineffective, AX-Glc caused appearance of condensed and disorganized stress fibers in both cell types after 24 h of treatment (Figure 3B) that was further pronounced after 48 h (data not shown). Importantly, the effects on ACs were less severe as only a small fraction were affected and cell shape was largely conserved after 24 h AX-Glc, whereas nearly all PCs displayed significant F-actin disruption and chaotic cell shape changes resulting in very long and thin processes (Figure 3B). These results underline our previous observation that both EC cell line and primary cultures are sensitive to O_2_ deprivation, whereas ACs and PCs can tolerate prolonged and severe O_2_ deprivation.

### Only ACs and PCs adapt their proliferation during O_2_ deprivation and ischemia

**Figure 4:**
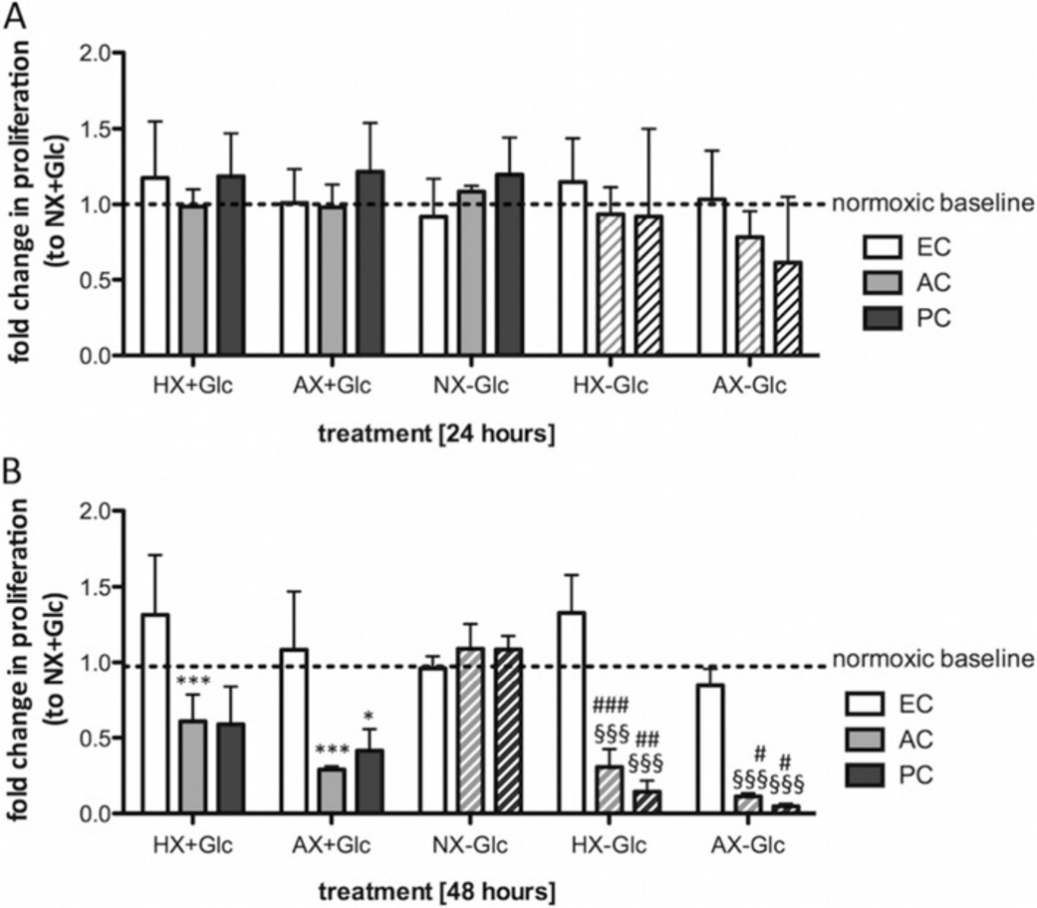
**Adaptation of proliferation by barrier associated cells during hypoxic and ischemic insults.** Proliferation of primary ECs, ACs and PCs was analyzed using BrdU incorporation assay after exposure to NX, HX and AX in presence (solid bars) and absence (hatched bars) of glucose for 24 h **(A)** and 48 h **(B).** Data values were normalized to normoxic baseline (NX + Glc). N = 3-5. *P < 0.05, **P < 0.01, ***P < 0.001; 1-way ANOVA compared to normoxia + glucose. ^§§§^ *P* < 0.001; 1-way ANOVA compared to normoxia-glucose. ^#^ *P* < 0.05, ^##^ *P* < 0.01, ^###^ *P* < 0.001; 2-way ANOVA comparing + to - glucose. EC: primary endothelial cells, AC: primary astrocyte, PC: primary pericyte, NX: normoxia, HX: hypoxia, AX: near anoxia, +Glc: with glucose, -Glc: without glucose.

Modulation of proliferation is a crucial factor to survive hypoxic/ischemic events [29]. Proliferation of primary ECs, ACs and PCs was measured using a BrdU incorporation ELISA. Exposure for 24 h in presence or absence of glucose did not alter the proliferation of any of the cells (Figure 4A) although a trend towards reduced BrdU incorporation under AX-Glc was measured, albeit slightly stronger in PCs (Figure 4A). Overall AC and PC BrdU incorporation correlated inversely with injury severity at 48 h. Indeed prolonged oxygen deprivation for 48 h significantly reduced proliferation in both groups and substantial inhibition was observed after 48 h of OGD (Figure 4B). Again a trend of PCs being more affected by glucose deprivation than ACs was apparent. Surprisingly, OGD exposure even up to 48 h did little to suppress primary EC proliferation and similar effects were observed using the EC cell line (data not shown) suggesting ECs maintain their proliferative rate during injury conditions.

### Hypoxic/ischemic exposure differentially affects metabolic activity of BBB associated cells

**Figure 5:**
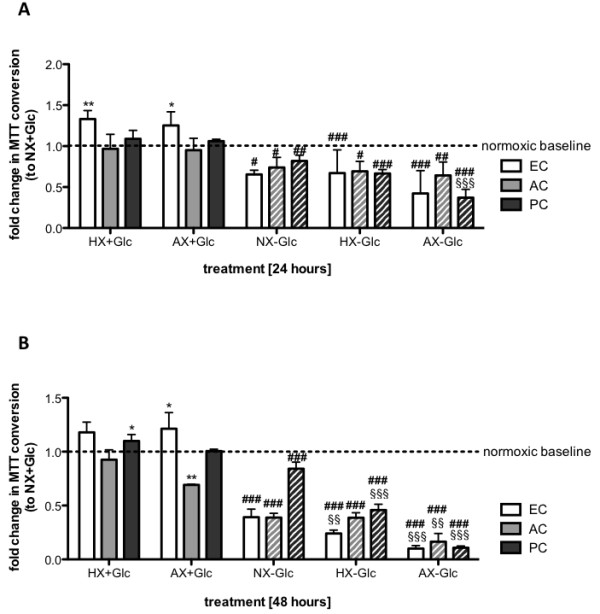
**Hypoxic/ischemic exposure differentially modulates metabolic activity of EC and perivascular cells.** Viability of primary ECs, ACs and PCs was measured by MTT conversion after exposure to NX, HX and AX in presence (solid bars) and absence (hatched bars) of glucose for 24 h **(A)** and 48 h **(B)**. Data was normalized to normoxic baseline (NX + Glc). N = 3-5. **P < 0.01, ***P < 0.001; 1-way ANOVA compared to normoxia + glucose. ^§§^ *P* < 0.01, ^§§§^ *P* < 0.001; 1-way ANOVA compared to normoxia-glucose. ^#^ *P* < 0.05, ^##^ *P* < 0.01, ^###^ *P* < 0.001; 2-way ANOVA comparing + to - glucose. EC: primary endothelial cell, AC: astrocyte, PC: pericyte, NX: normoxia, HX: hypoxia, AX: near anoxia, +Glc: with glucose, -Glc: without glucose.

To assess cellular viability, we measured metabolic activity using the MTT assay that allows overall evaluation of the degree of cellular impairment. After 24 h of HX or AX exposure, the metabolic activity of all cells remained unchanged, and even moderately increased in primary ECs (Figure 5A). Additional glucose withdrawal lowered the metabolic activity of all cells by 20-40% although AC performed slightly better in AX-Glc. In the presence of glucose, with the exception of reduced activity of AC in AX, prolonged treatment for 48 h had no effect on the different cells (Figure 5B). Interestingly, during prolonged normoxic glucose withdrawal PCs were significantly less affected than ACs and primary ECs that lost more than 50% of their initial activity. However, 48 h of HX-Glc gave similar degrees of AC and PC metabolic impairment that was accentuated in AX-Glc. It is noteworthy that we observed the RBE4 cell line to be significantly more sensitive than primary EC with their metabolic activity severely reduced by about 40% after 24 h HX or AX exposure and up to 70% after 48h (data not shown).

### Differential regulation of autophagy and cell death in BBB associated cells

**Figure 6:**
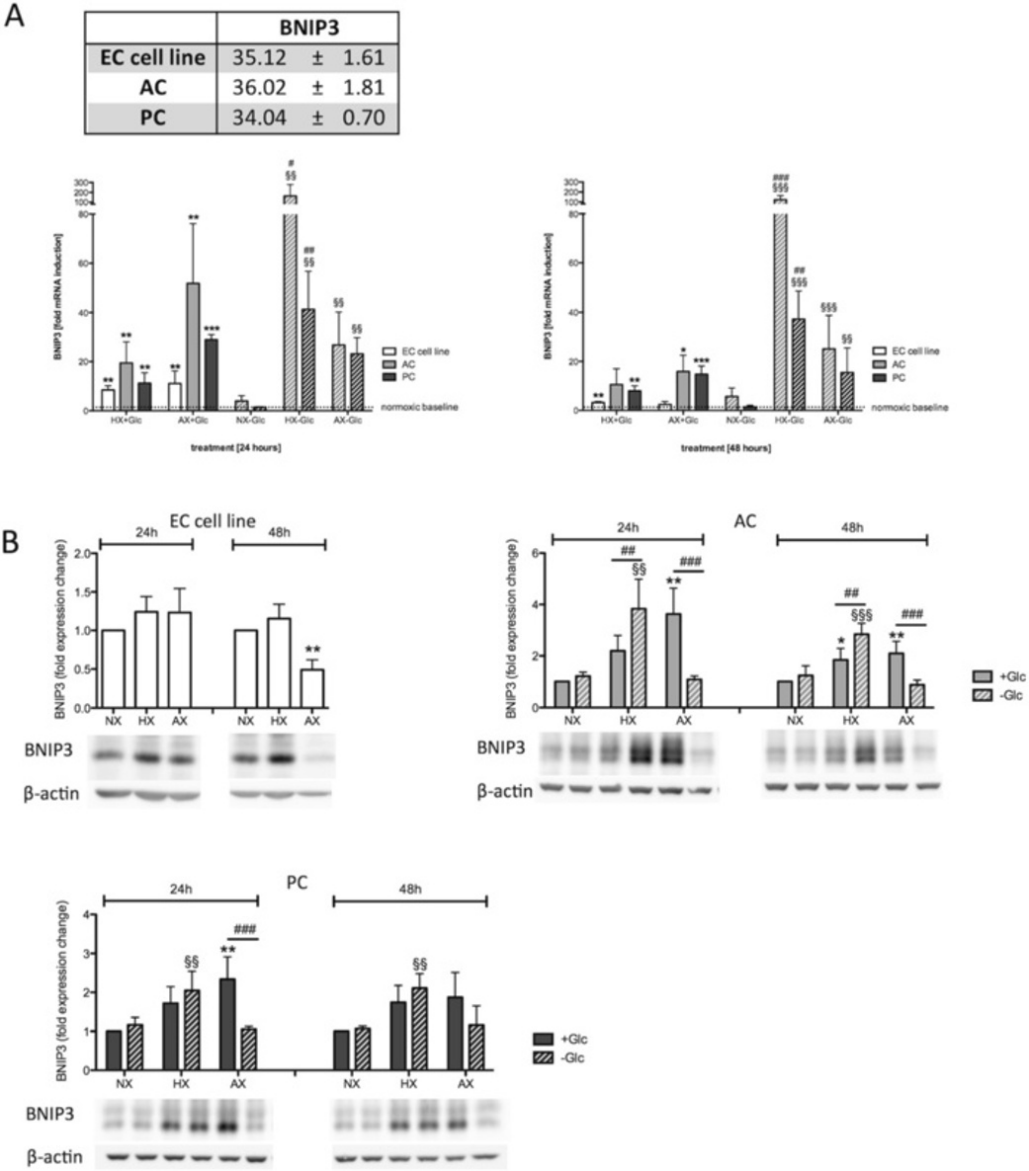
**Differential regulation of BNIP3 expression in barrier associated cells.** BNIP3 mRNA and protein expression was measured after 24 h and 48 h exposure to HX, AX and OGD and compared to NX. **(A)** Table of basal normoxic expression of BNIP3 showing threshold cycles (CT) ± standard deviation (upper panel). Lower panels BNIP3 mRNA expression after 24 h (left) and 48 h (right) of treatment. **(B)** Densitometric quantification of BNIP3 protein expression (upper panel) and representative Western blots (lower panels). N = 4. *P < 0.05, **P < 0.01, ***P < 0.001; 1-way ANOVA compared to normoxia + glucose. ^§^ *P* < 0.05, ^§§^ *P* < 0.01, ^§§§^ *P* < 0.001; 1-way ANOVA compared to normoxia-glucose. ^#^ *P* < 0.05, ^##^ *P* < 0.01, ^###^ *P* < 0.001; 2-way ANOVA comparing + to - glucose treatment. EC cell line: RBE4, AC: astrocyte, PC: pericyte, NX: normoxia, HX: hypoxia, AX: near anoxia, +Glc: with glucose, -Glc: without glucose.

**Figure 7:**
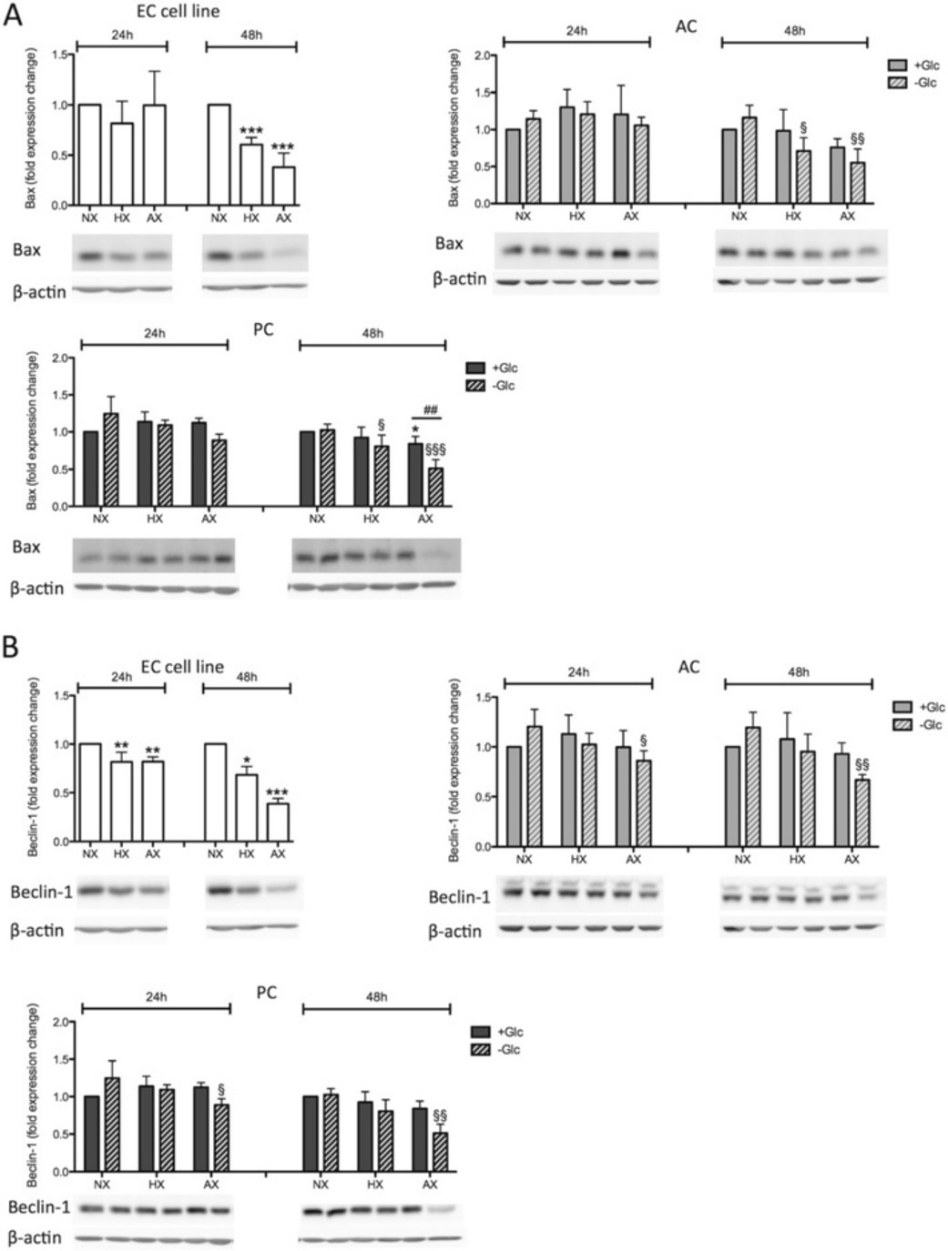
**Prolonged and severe hypoxic/ischemic treatment decreases Bax and Beclin-1 expression.** Densitometric quantification of Bax **(A)** and Beclin-1 **(B)** protein expression (upper panel) and representative Western Blots (lower panels) after 24 h and 48 h of O_2_ deprivation and ischemic treatment in EC cell line, ACs and PCs. N = 4. *P < 0.05, **P < 0.01, ***P < 0.001; 1-way ANOVA compared to normoxia + glucose. ^§^ *P* < 0.05, ^§§^ *P* < 0.01, ^§§§^ *P* < 0.001; 1-way ANOVA compared to normoxia-glucose. ^#^ *P* < 0.05, ^##^ *P* < 0.01, ^###^ *P* < 0.001; 2-way ANOVA comparing + to - glucose treatment. EC cell line: RBE4, AC: astrocyte, PC: pericyte, NX: normoxia, HX: hypoxia, AX: near anoxia, +Glc: with glucose, -Glc: without glucose.

**Figure 8:**
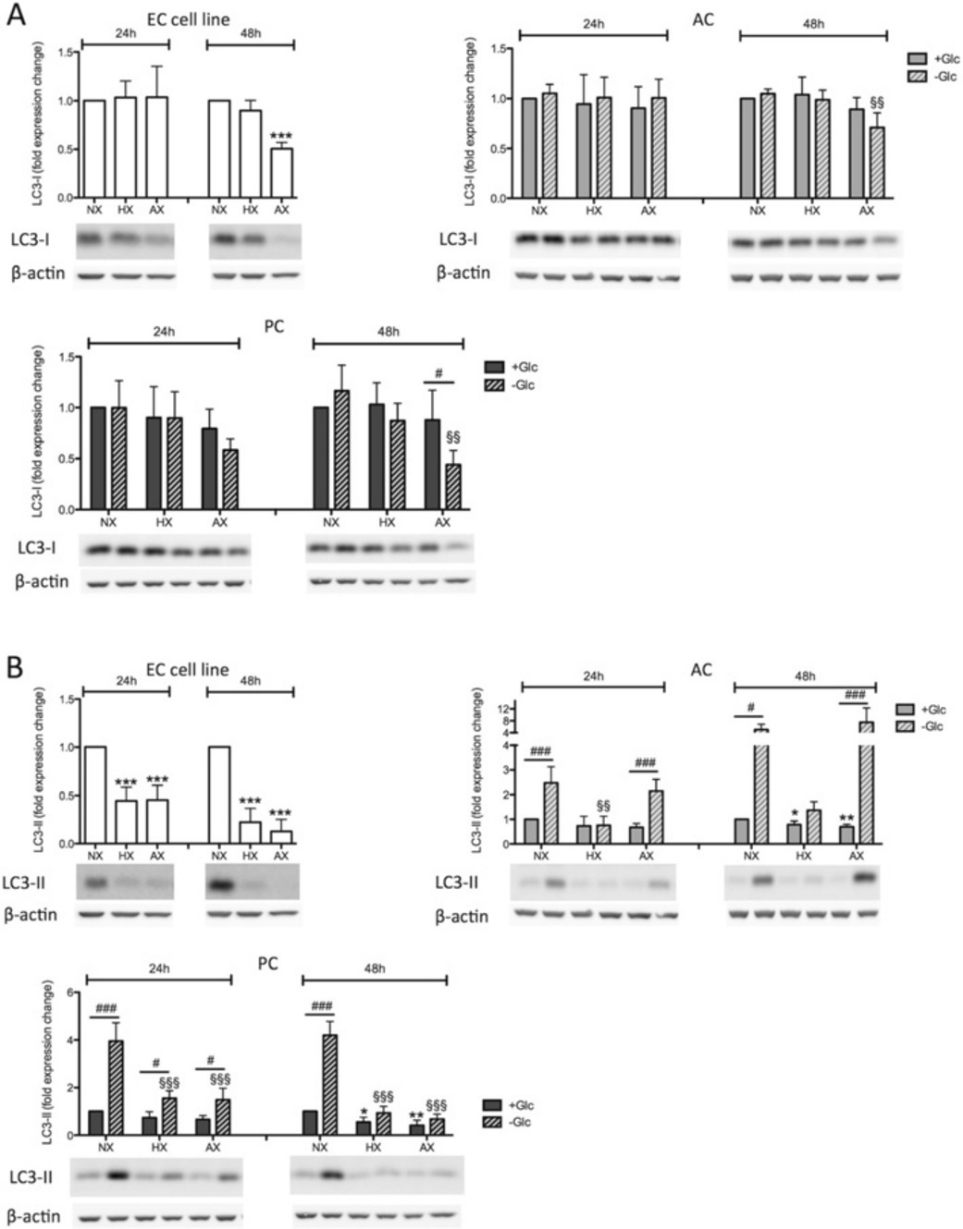
**Cell-specific regulation of LC3-II expression in barrier associated cells.** Representative Western Blots of LC3-I **(A)** and LC3-II **(B)** expression in ECs, ACs and PCs are shown in lower panels, upper panels represent graphs of densitometric analysis. Cells were exposed to NX, HX and AX in presence and absence of glucose for 24 h and 48 h. N = 4. *P < 0.05, **P < 0.01, ***P < 0.001; 1-way ANOVA compared to normoxia + glucose. ^§^ *P* < 0.05, ^§§^ *P* < 0.01, ^§§§^ *P* < 0.001; 1-way ANOVA compared to normoxia-glucose. ^#^ *P* < 0.05, ^##^ *P* < 0.01, ^###^ *P* < 0.001; 2-way ANOVA comparing + to - glucose treatment. EC cell line: RBE4, AC: astrocyte, PC: pericyte, NX: normoxia, HX: hypoxia, AX: near anoxia, +Glc: with glucose, -Glc: without glucose.

The above assays highlighted interesting differences in cell status and viability during different injury paradigms. However, as these assays do not allow direct conclusion of survival and cell death pathway activation, prominent apoptotic and autophagy markers were analyzed (Figures 6, 7 and 8). Since BNIP3 (BCL2/adenovirus E1B 19 kDa protein-interacting protein 3) is a HIF-1 target gene implicated in regulation of survival promoting autophagy, autophagy preceding cell death, as well as apoptosis and necrosis [30] its mRNA and protein expression levels were analyzed by qPCR and Western blot respectively (Figure 6). All three cell types expressed very low basal levels and upregulated BNIP3 transcription in response to O_2_ deprivation although their fold induction and temporal expression profiles differed strongly (Figure 6A). An initial 8–10 fold increase in EC BNIP3 mRNA after 24 h of HX and AX was measured but this was down-regulated after prolonged exposure to a 3-fold induction (Figure 6A). In total contrast, PCs and particularly ACs were highly responsive showing a severity dependent-induction that was largely evident during HX-Glc (Figure 6A). BNIP3 protein levels in the EC cell line showed a similar trend at 24 h with a significant reduction after 48 h of AX (Figure 6B). AC and PC protein levels correlated well with the qPCR induction profiles and the reduced fold-change induction of BNIP3 protein in PCs compared to ACs was also detected.

The consequences of increased hypoxic BNIP3 expression are controversial as both cell death/apoptosis promoting [30] and survival-promoting functions via induction of autophagy have been reported [31]. Cell death promoting functions of BNIP3 have been attributed to activation of Bax (Bcl-2 associated protein X) - a crucial regulator of mitochondria-dependent apoptosis that forms pores in the outer mitochondrial membrane allowing release of apoptotic proteins such as cytochrome C [32]. However, we did not observe Bax induction in any of the cells, and levels stayed constant up to 24 h of O_2_ deprivation and/or OGD (Figure 7A). Furthermore prolonged treatment for 48 h significantly reduced Bax levels in all cells suggesting that in our model BNIP3 does not induce Bax-mediated signaling. BNIP3 also regulates autophagy via the activation of Beclin-1 with subsequent formation of LC3-II [31]. Beclin-1 participates in early autophagosome formation by coordinating localization of autophagic proteins and membrane trafficking [33]. Surprisingly, similar to Bax we did not observe increased Beclin-1 expression in any of the cell types during injury, but a reduction in total protein levels particularly at AX-Glc treatments for ACs and PCs and at all hypoxic/anoxic conditions for the EC cell line (Figure 7B).

To further investigate this intriguing observation, we measured levels of LC3 (microtubule-associated protein light chain 3), a central protein of the canonical autophagy machinery and a down-stream target of Beclin-1 [33]. LC3 exists in 2 isoforms with LC3-I having a cytoplasmic localization whereas LC3-II, through conjugation with phosphatidylethanolamine (PE), localizes at membranes of autophagosomes and therefore its protein levels reflect the formation of autophagosomes [34]. In all cell types LC3-I levels were only significantly affected after prolonged exposure and severe insults, although PCs already showed a clear trend of reduced levels after 24 h (Figure 8A). In complete contrast, interesting cell-type specific LC3-II profiles were observed. Correlating well with the effects observed for BNIP3 expression, the EC cell line had significantly down-regulated LC3-II protein levels suggesting inhibition of autophagy during oxygen deprivation (Figure 8B). In presence of glucose, ACs and PCs only showed minor changes in LC3-II levels during O_2_ deprivation with a mild but significant reduction at 48 h (Figure 8B). However, an OGD-dependent increase in LC3-II protein levels was observed in both ACs and PCs (Figure 8B).

## Discussion

The BBB plays a pivotal role in supporting proper brain function and is modulated by signaling inputs of surrounding ACs and PCs to maintain its unique properties and function [3, 5, 19]. Similarly, ACs and PCs also modulate BBB tightness during hypoxic and ischemic insults, however, the critical factors mediating cell-specific responses as well as their functional consequences on BBB integrity remain a topic of debate [5–7, 12, 35]. One reason for this is that our understanding of individual BBB cellular component responses in terms of proliferation, survival, target gene induction and barrier modulation during injury is very limited. This study is the first to directly compare hypoxic and ischemic responses of all three major cells types at the BBB and to address various aspects of cellular adaptation and survival. We show that the BBB-associated cells differ considerably in sensitivity to hypoxic and ischemic insults. All our data points to the fact that endothelial cells experience O_2_ and glucose deprivation more severely than the other cell types and show a higher susceptibility. Two publications by Ceruti *et al.* and Redzic *et al.* recently compared survival of primary rat ECs, ACs and PCs under OGD (0% O_2_) conditions and similarly concluded that ECs are more susceptible than ACs and PCs [36, 37]. Importantly, our data further suggests that ACs tolerate ischemic injury better than PCs. In this regard, it is noteworthy that the differential tolerance of these cells correlates well with the degree of O_2_ they experience under physiological conditions *in vivo*. Indeed, in brain capillaries an O_2_ content of roughly 8% (pO_2_ 58 mm Hg) has been recorded [38], whereas O_2_ concentrations in the brain parenchyma are about 4% (pO_2_ 35 mm Hg) [39] thus based on their localization ECs are accustomed to higher physiological O_2_ concentrations than PCs and ACs.

The HIF-1 signaling pathway crucially controls hypoxic/ischemic gene expression for cell survival and energy metabolism but also the expression of genes regulating apoptosis and autophagy [18, 19]. Growing evidence suggests that the severity as well as the duration of an insult play a crucial role in the pathways activated [5, 6, 16, 17]. Indeed, we observed the more sensitive ECs stabilize 3–5 times more HIF-1α protein than the relatively resistant ACs and PCs, suggesting that HIF-1 induction correlates to sensitivity and may determine functional outcome for the different cells. In addition, HIF-1α was rapidly induced and O_2_ deprivation severity did not alter the amount of HIF-1α stabilized in either ECs or PCs. This “stereotype” maximal HIF-1α response of ECs and PCs to both mild and more severe insults indicates an inability to respond in a more fine-tuned manner as observed with ACs. The ECs also sense low O_2_ very rapidly and respond with a strong short lasting HIF-1α stabilization highlighting a greater responsiveness to O_2_ deprivation than the perivascular cells. Other studies have also shown that HIF-1α induction is strongly cell-type dependent. A study by Bracken *et al.* showed that whereas some cells readily stabilize HIF-1α at 5% O_2_, others require O_2_ levels below 1% and that HIF-1α transactivation is also dependent on the severity of the hypoxic insults in different cell types [40]. As a direct consequence of differential HIF-1α stabilization, mRNA levels of select HIF-1 target genes were also cell-specifically regulated. Pronounced induction of VEGF (particularly during mild hypoxia) and GLUT-1 in EC compared to the other cell types correlated well with substantial HIF-1α stabilization, again underlining their strong responsiveness to hypoxic events. Interestingly, despite early HIF-1α stabilization similar to ECs, PCs did not induce VEGF expression in comparable strength. Indeed, reduced HIF-1α stabilization in PCs compared to ECs suggests the amount of HIF-1 accounts for the differential regulation of these genes. In contrast, PHD2 and BNIP3 induction levels were reduced in ECs compared to ACs and PCs although both are HIF-1 target genes, suggestive of either a cell type-specific regulation/activation or a severity-dependent regulation of HIF-1 target gene expression. In agreement it was shown previously that basal mRNA expression of hypoxia-inducible genes varies considerably amongst cell types and that cell-type specific induction of target genes occurs [41]. Similar to our conclusions, the authors correlated the amount of HIF-1α transcripts and protein abundance to the fold induction of hypoxia responsive genes and, importantly, to the susceptibility of the cells to hypoxic injury [41].

Since appropriate adaptation of energy metabolism is a crucial factor for surviving injurious events like O_2_ deprivation and ischemia, transcriptional regulation of the glucose transporter GLUT-1 was of particular interest as glucose represents the major brain energy source [42]. During O_2_ deprivation, glycolysis is crucial for ATP production and our observation of strong induction of GLUT-1 by EC during O_2_ deprivation agrees with literature showing that cerebral vascular glucose consumption and transport increases in response to hypoxia [43]. This raises the question whether ECs are more dependent on glucose uptake to feed their ATP consumption during O_2_ deprivation than ACs and PCs. Possible reasons for this can either be that PCs and ACs have different energy metabolism and/or less reliance on glucose *per se* due to efficient use of alternate energy sources. Interestingly GLUT-1 induction was lower in ACs than PCs underlying the exceedingly versatile metabolism of ACs, i.e. the ability to exhibit high rates of oxidative metabolism and glycolysis, as well as the use of alternate energy fuels such as glycogen or glutamate [1, 44, 45]. Indeed it has long been known that ACs are the major glycogen store within the brain, whereas capillary endothelial cells and PCs have only minor reserves [46, 47]. Although further study is required, it is possible that PCs could employ mechanisms similar to ACs to support their resistance to hypoxic/ischemic injuries.

A more global interpretation of cellular well-being was obtained by monitoring actin cytoskeletal architecture and metabolic activity using MTT assay - both of which emphasized swift alteration and impairment of ECs. Notably, rapid injury-mediated cytoskeletal reorganisation in ECs coincided with loss of BBB function under similar conditions in our previous studies [2, 6]. Interestingly, changes in cytoskeletal structure preceded metabolic suppression in all cell types but marked alterations in AC and PC organisation occurred only during prolonged OGD correlating well with cell survival and highlighting their ability to cope well with hypoxia/ischemia. Similar conclusions can be drawn from our BrdU proliferation assays showing that both cells adapted their proliferation depending on duration as well as insult severity to similar levels - likely a crucial response to reduce energy consumption and foster cell survival. In line with this, a microarray study showed that many hypoxia down-regulated genes are linked to proliferation [41]. In contrast, increased AC proliferation during hypoxia and ischemia has also been reported *in vivo* and *in vitro* during acute (6 h) OGD and hypoxic exposure [48–50]. We did not observe increased AC proliferation under any condition probably due to the more chronic time points selected suggesting time-dependency for the different severities. Perplexingly, despite being more impaired than ACs or PCs, ECs maintained their proliferation regardless of the severity of injury. Maintaining high proliferation, and therefore high energy consumption, during adverse conditions only confounds cellular adaptation and has an overall negative impact on survival. Although it remains somewhat a mystery why the ECs do not suppress/reduce proliferation, a link with angiogenesis-mediated responses during oxygen deprivation could be suggested [5].

The HIF-1 target gene BNIP3 represents a point of intersection in regulation of cell viability during O_2_ deprivation. For BNIP3, both pro-apoptotic/cell death promoting functions via Bax activation [30], and survival-promoting functions via autophagy-related proteins including Beclin-1 and LC3-II [31], have been observed. Our data suggest that BNIP3 does not promote Bax-mediated cell death in our model system but observations of increased LC3-II expression points towards BNIP3-mediated induction of autophagy. This observation is further supported by the resistant ACs and PCs showing strong BNIP3 expression, whereas the more sensitive EC cells did not. Unexpectedly, the expression profile of LC3-II did not match that of BNIP3, indeed we frequently observed an inverse correlation. It is notable that LC3-II is suggested to be a good indicator of the number of autophagosomes formed but not necessarily autophagic flux, as LC3-II itself is degraded during autophagosome maturation [34]. For this reason, strong activation of autophagy can lead to low LC3-II levels because of its rapid degradation, which may then be misinterpreted as a lack of autophagy. Although our results point towards activation of autophagic pathways by ACs and PCs to modulate their cell survival, additional experiments are required to fully understand the underlying mechanism. Nevertheless it is tempting to speculate that autophagy may represent an adaptive mechanism of ACs and PCs to cope with hypoxic/ischemic injury by facilitating removal of damaged mitochondria and regeneration of ATP by catabolism of existing cytoplasmic precursors [51]. It remains unclear why brain ECs do not employ similar mechanisms in response to hypoxia although it must be noted that hypoxia-mediated autophagy induction in peripheral endothelial cells has been demonstrated [52]. We hypothesize that absence of hypoxic BNIP3 induction underlies insufficient activation of survival-promoting autophagy and contributes to increased EC sensitivity to hypoxic stress.

## Conclusion

This study confirms that BBB cell types vary considerably in their sensitivity to O_2_ deprivation and ischemia. We showed that on different levels of cellular responses ECs are more susceptible to O_2_ deprivation whereas ACs and PCs are very tolerant to sole O_2_ deprivation. It is also noteworthy that although the EC cell line proved to be more susceptible to injury compared to the primary EC cells, similar overall responses and pathway signaling mechanisms were seen in both. Our data further establishes that perivascular cells are predominantly glucose responsive with ACs withstanding extreme ischemic exposure (48 h, 0.2% O_2_) better than PCs. Overall we propose that the physiological oxygen concentrations experienced by cells underpin their sensitivity to insult. Since differential susceptibility can directly and/or indirectly impact BBB stability, a better understanding of such causative relationships is crucial to combat BBB disturbance during injury.

## Abbreviations

AC: Primary astrocyte
AX: Near anoxia
Bax: Bcl-2 associated protein X
BBB: Blood–brain barrier
BNIP3: BCL2/adenovirus E1B 19kDa protein-interacting protein 3
EC: Endothelial cell
Glc: Glucose
GLUT-1: Glucose transporter 1
HIF-1: Hypoxia inducible factor-1, HX hypoxia
LC3: Microtubule associated protein light chain 3
MMP: Matrix metalloproteinase
OGD: Oxygen-glucose deprivation
PC: Primary pericyte
PHD2: Prolyl hydroxylase 2
RBE4: Rat brain microvascular endothelial cell line
VEGF: Vascular endothelial growth factor.
